# In vitro immunotoxicity of important mycotoxins detected in commercial dry dog and cat food in relation to internal exposure

**DOI:** 10.1007/s12550-026-00651-3

**Published:** 2026-05-18

**Authors:** Sven Dänicke, Susanne Kersten, Janine Saltzmann, Nicola Mickenautsch, Michael Sulyok, Dian Schatzmayr, Barbara Doupovec, Nadine Paßlack

**Affiliations:** 1 Institut für Tierernährung, Friedrich-Loeffler-Institut (FLI), Braunschweig, Germany; 2 Institute of Bioanalytics and Agro-Metabolomics, Department of Agricultural Sciences, BOKU University, Tulln, Austria; 3 dsm-firmenich, Animal Nutrition & Health R&D Center Tulln, Tulln, Austria; 4 Lehrstuhl für Tierernährung und Diätetik, Ludwig-Maximilians-Universität München, Oberschleißheim, Germany

**Keywords:** Dog, Cat, Pet food, PBMC, Cytotoxicity, Mycotoxins

## Abstract

A screening of commercial dog and cat foods revealed considerable variation in metabolite profiles across feed categories, with standard feeds generally showing higher concentrations of *Fusarium* metabolites, phytoestrogens, and plant metabolites than specialized senior or intestinal diets. Concentrations of aflatoxin B1 (AFB1), deoxynivalenol (DON) DON, zearalenone (ZEN), ochratoxin A (OTA), fumonisin B1 + 2 (FB1 + FB2), and T-2/HT-2 toxins remained below existing guidance values and maximum limits. In vitro assays using peripheral blood mononuclear cells (PBMCs) from dogs and cats demonstrated marked interspecies differences in mycotoxin sensitivity. Feline PBMCs were more susceptible to proliferation inhibition by DON and especially ZEN than canine PBMCs, consistent with known species-specific differences in xenobiotic metabolism. The potency ranking of tested compounds in dogs was in line with reports from other species, with T-2 toxin showing the highest cytotoxicity (EC50 < 0.1 µM). Analysis of feed and blood samples indicated that systemic exposure to DON and ZEN under practical feeding conditions was several orders of magnitude below in vitro EC50 values, suggesting limited direct cytotoxic risk. Nonetheless, the presence of multiple mycotoxins in all feed categories underscores the need for continued surveillance and further studies linking feed composition, toxin occurrence, and toxicological thresholds to better inform companion animal health risk assessments.

## Introduction

Commercial pet foods for dogs and cats often contain substantial amounts of plant-based ingredients, particularly cereals, which are susceptible to contamination with mycotoxins (Böhm et al. 2010; Leung et al. 2006; Witaszak et al. 2019, 2020; Zhou et al. 2024). In contrast, animal-derived feed components are considered a negligible source of contamination, as the carry-over of most relevant mycotoxins into animal tissues is minimal (MacLachlan 2011). This is supported by analytical studies showing that cereal-free dog foods contained no detectable levels of deoxynivalenol (DON), zearalenone (ZEN), or fumonisins B1 and B2 (FB1, FB2), whereas these mycotoxins were present in formulations containing cereals (Tegzes et al. 2019). These findings demonstrate that not only herbivorous species but also dogs and cats are exposed to mycotoxins, with the degree of external exposure largely depending on both the proportion and the contamination level of plant or cereal-based ingredients.

Different cereal types may pose varying risks, not only in terms of individual mycotoxin contamination but also with respect to characteristic co-contamination patterns involving multiple mycotoxins. Accordingly, specific guidance values for DON, ZEN, and FB1 + FB2 have been established for maize and maize-derived feeds. While the European Union sets maximum levels for aflatoxin B1 (European Union, 2002) and indicative guidance values for DON, ZEN, ochratoxin A (OTA), and T-2 + HT-2 toxins in pet foods (European Commission, 2013a, 2013b, 2006), toxicological data on their immunotoxic and cytotoxic potential in companion animals remain limited. In particular, information on cellular sensitivity to these mycotoxins - e.g., effective concentrations leading to 50% inhibition of viability or proliferation (EC50) - is scarce for dogs and cats.

Primary peripheral blood mononuclear cells (PBMCs) are widely used as an in vitro test system to evaluate cytotoxic and immunotoxic effects of mycotoxins in various species, as they represent a heterogeneous and immunologically relevant cell population. Dose-dependent toxic effects, including changes in cell viability and proliferation, can be reliably assessed using standardized metabolic assays such as the MTT test, which quantifies mitochondrial activity as a proxy for cellular metabolic function. However, to interpret such in vitro-derived EC50 values in a biologically relevant context, they must be compared to internal exposure levels under practical feeding conditions. Internal exposure can be estimated from blood concentrations of parent mycotoxins and their biotransformation products (metabolites), but such data are extremely limited for dogs and cats.

The present study aimed to provide a comprehensive overview of feed-derived metabolites, including mycotoxins, in selected commercial dog and cat food (Screening). Among the various mycotoxins, DON and ZEN were further investigated as key contaminants, with a particular focus on the relationship between external and internal exposure. External exposure was assessed by analyzing DON and ZEN concentrations in feed samples, while internal exposure was evaluated through the quantification of these toxins and their known metabolites in blood. This approach aimed at a direct comparison between feed contamination levels and systemic availability. Furthermore, internal exposure levels were related to EC50 values obtained from PBMC-based cytotoxicity assays, in order to provide an initial toxicokinetic–toxicodynamic (TK–TD) characterization of the associated mycotoxin-related risks in companion animals.

## Materials and methods

### Sampling of feed and blood for screening

#### Feed

According to their label declarations, the available commercial feed samples were assigned to five categories: “all dog feed” (*n* = 2), “cat indoor feed” (*n* = 1), “cat senior feed” (*n* = 1), “cat intestinal feed” (*n* = 1) and “other cat feed” (*n* = 9). The composition of these feeds was as follows: The all dog feed consisted of plant by-products, meat and animal by-products, oils and fats (including 0.9% fish oil), fruits, minerals, and seeds. Corn starch served as the main carbohydrate source, while hydrolyzed chicken liver (26%) was used as the primary protein source. The cat indoor feed contained meat and animal by-products (lamb meal and turkey meal), cereals (maize and rice), oils and fats, plant by-products (dried beet pulp), eggs and egg products (whole egg powder), yeast (dried), minerals, algae (*Ascophyllum nodosum*, dried), seeds (flaxseed), herbs (dried), and chicory root (dried, source of inulin, 0.1%). The cat senior feed consisted of meat and animal by-products (poultry meal and lamb meal), cereals (maize and rice), oils and fats, fish and fish by-products (salmon meal), plant by-products (dried beet pulp), eggs and egg products (whole egg powder), minerals, yeast (dried), algae (*Ascophyllum nodosum*, dried), seeds (flaxseed), herbs (dried), chicory root (dried, source of inulin, 0.1%), extracted yeast (dried, source of mannan oligosaccharides [MOS], 0.1%), and green-lipped mussel (*Perna canaliculus*, dried, 0.05%). The cat intestinal feed contained vegetables (sweet potato, dried), meat and animal by-products (duck meal), oils and fats, plant by-products (cellulose), chicory root (dried, source of inulin, 0.2%), minerals, and seeds (flaxseed).

#### Blood samples

Residual blood plasma and serum samples of approved animal experiments (approval numbers: A 0159/18 (LAGeSo, Berlin, Germany) in combination with GZ 2347-A3-3–2018 (local authority in Brandenburg, Germany); G0303/16 (LAGeSo, Berlin, Germany) were used for the screening analyses. These samples were stored at −80 °C and were no longer required for analyses of the respective animal trials.

### In vitro cytotoxicity study

#### Experimental design

In the in vitro experiments, PBMCs from dogs and cats were exposed to various concentrations of selected mycotoxins, with and without stimulation by concanavalin A (Con A). For the evaluation of all dose–response relationships, the control condition without toxin addition (0 µM) was used as the reference, separately for each species.

Canine PBMCs were treated with DON at concentrations of 0.1, 0.5, 1.0, 1.5, 2.0, and 10.0 µM. De-epoxy-DON (de-DON) was applied at 0.1, 1.0, 5.0, 10.0, 50.0, and 100.0 µM. ZEN was tested at 0.1, 0.5, 1.0, 10.0, 50.0, 75.0, and 100.0 µM, while α-zearalenol (α-ZEL) was examined at 0.1, 0.5, 1.0, 10.0, 50.0, 75.0, and 100.0 µM. For T-2 toxin (T-2), concentrations of 0.1, 0.5, 1.0, 1.5, 2.0, and 10.0 µM were applied. 3-acetyl-DON (3-AC DON) was tested in the range of 0.1, 0.5, 1.0, 1.5, 2.0, and 10.0 µM.

Feline PBMCs were treated with DON at 0.1, 0.5, 1.0, 1.5, 2.0, and 10.0 µM, and with ZEN at 0.1, 0.5, 1.0, 10.0, 50.0, 75.0, and 100.0 µM. The restriction to these two mycotoxins was due to the limited availability of feline PBMCs, which resulted from comparatively small blood volumes and lower PBMC yields after Biocoll gradient separation.

All test compounds were initially dissolved in appropriate solvents depending on their physicochemical properties. DON, ZEN, α-ZEL, T-2 toxin, and 3-AC DON were prepared in 100% DMSO. De-DON was supplied in acetonitrile; the solvent was removed prior to use, and the compound was reconstituted in medium containing 13% DMSO. All compounds were subsequently diluted in cell culture medium to the respective final concentrations. The final solvent concentration was adjusted to 2.7% DMSO in all treatments, including the control (0 µM), which served as vehicle control.

#### Blood samples

The blood collection for the PBMC isolation from fresh blood samples for the in vitro cytotoxicity trials was approved by the relevant local ethics committee (GI 18/17 Nr. A 11/2020 Transfer, Regierungspräsidium Giessen, Germany). The sampling was performed in the fasting state of the animals and by puncture of the *Vena cephalica antebrachii*.

The samples used for screening analyses were independent from those used for the in vitro cytotoxicity experiments and originated from separate animal studies with different approval numbers.

#### Isolation and in vitro culture of PBMCs

PBMCs were isolated from freshly collected heparinized blood. For this purpose, the blood samples were diluted 1:1 with phosphate-buffered saline (PBS) and subjected to density gradient centrifugation using Biocoll separation solution (Biochrome AG, Berlin, Germany). The subsequent steps followed the protocol described by (Goyarts et al. 2006).

Cell viability was assessed using the trypan blue exclusion method. Viable PBMCs were seeded at a density of 1 × 10⁵ cells per 200 µL in 96-well culture plates. Test compounds were added immediately after seeding. Cells were then incubated at 37 °C with 5% CO₂ for 72 h. Cells were exposed to increasing concentrations of the test compounds, either without stimulation or in the presence of the mitogen ConA. After incubation, the plates were centrifuged and 100 µL of the supernatant removed. Subsequently, 10 µL of MTT solution (3-[4,5-dimethylthiazol-2-yl]−2,5-diphenyltetrazolium bromide) were added, and cells were incubated for an additional 4 h. The resulting formazan crystals were solubilized with 100 µL of 0.01 N HCl/SDS solution at room temperature for approximately 12 h. Finally, absorbance was measured at 570 nm.

### Analyses

#### Multi-toxin/metabolite analyses of dog and cat feed samples

Samples were homogenized with a Robot Coupe Blixer 4 (Robot Coupe, Montceau-en-Bourgogne, France). 5 g of homogenized material were weighed into 50 mL Falcon tubes and extracted using 20 mL of acetonitrile/water/acetic acid 79:20:1, v/v/v for 90 min on a GFL 3017rotary shaker (GFL; Burgwedel, Germany). Afterwards the tubes were placed in upright position to sediment particles and 500 µL of the clear supernatants were transferred into HPLC vials and diluted with 500 µL of acetonitrile/water/acetic acid 20:79:1, v/v/v. After appropriate mixing, 5 µL of the diluted extracts were injected into the LC–MS/MS system without further pre-treatment.

Mycotoxin analysis was carried out using a 1290 Series HPLC System (Agilent, Waldbronn, Germany) coupled to a QTrap 5500 LC-MS/MS System (Applied Biosystems SCIEX, Framingham, MA) equipped with Turbo Ion Spray electrospray ionization source. Chromatographic separation was performed at 25 °C on a Gemini^®^ C18-column, 150 × 4.6 mm i.d., 5 μm particle size, equipped with a C18 4 × 3 mm i.d. security guard cartridge (Phenomenex, Torrance, CA, US) running an acidified methanol/water gradient containing 5 mM ammonium acetate. ESI-MS/MS data was acquired in the scheduled multiple reaction monitoring mode both in positive and negative polarity in two separate chromatographic runs. The detection window width was 40 and 46 s in the positive and negative ionization mode, respectively. The target cycle time was 1400 msec and the MS pause time was 3 msec. Compound dependent MS/MS parameters and source of reference standards are listed in Sulyok et al. (2024).

Confirmation of positive metabolite identification was obtained by acquiring two MS/MS signals per analyte which yielded 4.0 identification points according to the European Commission decision 2002/657. In addition, retention time and ion ratio had to agree to the related values of authentic standards within 0.03 min and 30% rel., respectively. Quantitation was based on external calibration using serial dilutions of a multi-analyte stock solution. Results were corrected for apparent recoveries determined for maize-based chicken feed (Steiner et al., 2020). The performance of the method is verified on a continuous basis by participation in a proficiency testing scheme (BIPEA, Genneviliers, France) with 128 out of 133 results submitted for dog feed, all 103 results submitted for cat feed and > 96% of the > 2500 results submitted overall exhibiting a z-score of −2 < z < 2. Limits of detection and limits of quantification were determined according to the EURACHEM guide and are listed in Sulyok et al. (2024).

#### Mycotoxin residues in canine and feline blood plasma and serum samples

Serum and plasma samples were analysed using a multi-method described in detail by Brezina et al. (2014), which included ZEN and its metabolites (α-zearalenol (α-ZEL), β-zearalenol (β-ZEL), zearalenone (ZAN), α-zearalanol (α-ZAL) and β-zearalanol (β-ZAL), as well as DON and de-DON. Each sample (500 µL) was spiked with 20 µL of internal standards (250 ng/mL for ^13^C_18_-ZEN, α-ZEL-d_4_, β-ZEL-d_4_, α,β-ZAL-d_4_ and 500 ng/mL for ^13^C_15_-DON), 1 mL of a sodium acetate buffer solution with a pH of 5.5, and 20 µL of β-glucuronidase (type H-2 from *Helix pomatia*, Sigma-Aldrich, Steinheim, Germany). The samples were incubated overnight at 37 °C and then purified using Oasis HLB solid phase extraction cartridges (Waters, Milford, MA, USA). Analytes were determined using LC-MS/MS, consisting of an Agilent 1200 series liquid chromatograph (Agilent Technologies, Böblingen, Germany) coupled with a 4000 QTrap mass spectrometer (Applied Biosystems, Darmstadt, Germany) equipped with an electrospray ionisation (ESI) source. The limits of detection (LOD) and quantitation (LOQ) for each matrix are shown in Table 1. The results were not corrected for recovery, which ranged from 85 to 123%.

**Table 1:** Limits of detection (LOD, ng/mL) and limits of quantification (LOQ, ng/mL) of zearalenone, deoxynivalenol and their metabolites in analysed blood samples

Toxin	Dog serum		Dog plasma		Cat serum	
	LOD^a^	LOQ^b^	LOD^a^	LOQ^b^	LOD^a^	LOQ^b^
Zearalenone (ZEN)	0.31	1.03	0.24	0.80	0.02	0.07
α-zearalenol (α-ZEL)	4.79	15.97	4.21	14.05	0.43	1.42
β-zearalenol (β-ZEL)	0.44	1.47	0.17	0.57	8.66	28.86
Zearalanone (ZAN)	11.65	38.83	8.99	29.98	0.89	2.97
α-zearalanol (α-ZAL)	0.30	1.01	0.31	1.02	0.26	0.86
β-zearalanol (β-ZAL)	18.35	61.18	15.48	51.59	13.59	45.3
Deoxynivalenol (DON)	1.1	3.66	1.29	4.32	1.68	5.59
De-epoxy-deoxynivalenol (de-DON)	0.53	1.77	0.47	1.58	5.30	17.67

^a^ Limit of detection based on a S/N ratio of 3

^b^ Limit of quantification based on a S/N ratio of 10

####  Calculations and statistics

For each dose level, optical density (OD) values obtained from the MTT assay were normalized to the OD of the untreated control (without toxin) and expressed as percent inhibition, both in the absence and presence of ConA. To calculate the stimulation index (SI), the OD measured after stimulation with ConA was divided by the OD obtained without ConA.

The primary objective of the study was to identify suitable indicators of cytotoxicity for various toxins, with the EC50 serving as a key metric. To estimate EC50 and related parameters, data were initially fitted to a four-parameter logistic (4PL) model, assuming a sigmoidal dose–response relationship. When the observed data did not exhibit a sigmoidal pattern - particularly when the maximum effect was already reached at the lowest tested concentration - a broken-line (segmented) model was applied instead. This approach assumed that no further biological response occurred at higher concentrations beyond a certain threshold (breakpoint), and that any variability observed was attributable to technical or biological noise. Consequently, the broken-line model incorporated a second segment that was flat, representing a constant response and running parallel to the X-axis beyond the estimated breakpoint.

The four-parameter logistic (4PL) regression model:

$$\:\widehat{y}\left(x\right)=c+\frac{d-c}{1+\mathrm{exp}\left(b\cdot\:\left(\mathrm{log}x-\mathrm{log}e\right)\right)}$$

fits toxin concentrations (x) to inhibition (y) non-linearly and describes sigmoidal dose-response relationships using four interpretable parameters. The lower asymptote c represents the minimal response at low concentrations; the upper asymptote d indicates the maximal response at high concentrations. The inflection point e corresponds to the concentration at which the response is halfway between c and d, commonly referred to as the EC50. Parameter b defines the slope of the curve around the inflection point, reflecting how steeply the response changes with increasing concentration.

While the EC50 is included as a parameter (e) in the four-parameter logistic model during curve fitting, it does not directly correspond to the effective concentration that induces 50% of the maximal effect. Instead, true effective concentrations such as EC10 or EC50 are calculated post hoc based on the fitted curve, as the response values must first be normalized to a 0–100% scale. Thus, the effective concentration ECx​ corresponding to an effect level x (e.g., 10%, 50%, etc.) in the four-parameter logistic model is calculated as follows:

$$\:ECx=e\cdot\:\mathrm{exp}\left(\frac{1}{b}\cdot\:\mathrm{ln}\left(\frac{d-c}{y-c}-1\right)\right)$$

$$\:\mathrm{where}\hspace{1em}y=c+\frac{x}{100}\cdot\:\left(d-c\right)$$

The broken-line model (BLM) is defined as:

$$\hat{y}(x)=\begin{cases}\mathrm{intercept}+\mathrm{slope}\cdot x, & \text{if } x \leq \mathrm{breakpoint}\\\mathrm{constant}, & \text{if } x> \mathrm{breakpoint}\end{cases}$$

with

$$\:\mathrm{breakpoint}=\frac{\mathrm{constant}-\mathrm{intercept}}{\mathrm{slope}}$$

and fits toxin concentrations (x) to inhibition (y) around the breakpoint in a linear fashion. The intercept (on ordinate) is the starting value of the response at x = 0, the slope equals the linear increase of the response before the breakpoint, the constant is the plateau response after the breakpoint, and the breakpoint reflects the toxin concentration at which the model changes from a linear increase to a constant.

Heatmap grading was derived within each toxin group by comparing feed-specific medians to an overall N-weighted median across all feed types. Relative effects were calculated as log-ratios of medians and weighted by the square root of the sample size. Group-specific quantile thresholds (25th and 75th percentiles) were used to classify values as Lower, Neutral, or Higher.

All statistical analyses were conducted using R version 4.3.2 (Core Team 2023). Dose–response curves were modeled using a four-parameter logistic function (4PL) fitted *via* the function ***drm*** of the package ***drc*** (Ritz et al. 2015), whereas segmented (broken-line) models were implemented using a custom function based on numerical gradient optimization.

## Results

### Fungal and plant-derived metabolites in pet food

**Fig. 1:**
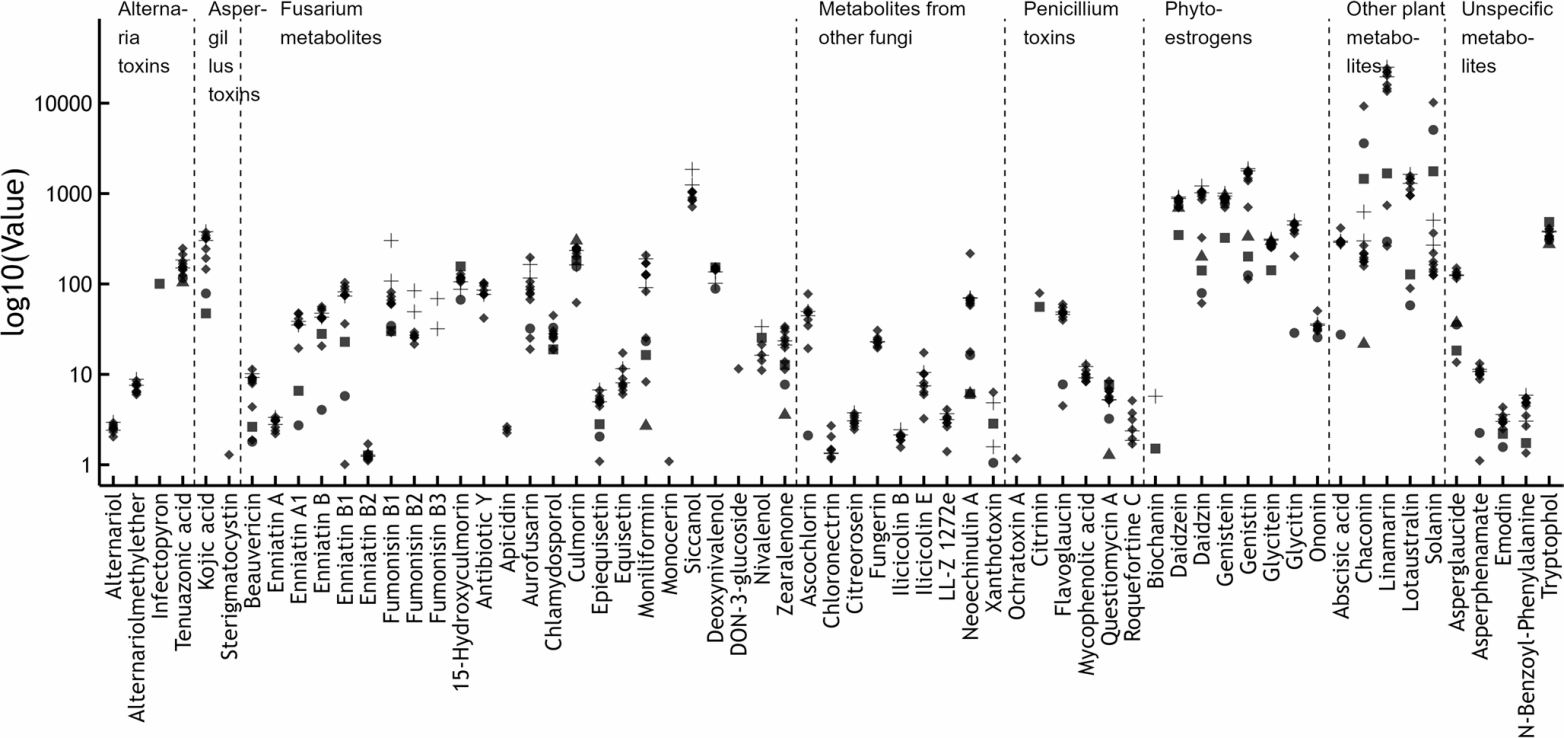
Multi-metabolite profiles of individual dog and cat food samples (µg/kg as fed). Feed categories are indicated by symbol type: circles (cat indoor), squares (cat senior), triangles (cat intestinal), diamonds (other cat), and crosses (all dog feed)

In total, 63 different analytes covering a wide range of mycotoxins (produced by *Fusarium*, *Aspergillus*, *Alternaria*, and *Penicillium* species), as well as plant and unspecific metabolites, were quantified (Fig. 1).

Among the most frequently detected and abundant fungal metabolites were siccanol (median concentration = 1030 µg/kg), culmorin and 15-hydroxyculmorin (median concentration = 235 µg/kg and 114 µg/kg), and DON (median concentration = 145 µg/kg). Some of these compounds, known to be produced by *Fusarium* species, were also selected for blood analysis of cats and dogs. A broad range of additional mycotoxins—including enniatins, beauvericin, fumonisins, and various *Alternaria* metabolites—was also detected, although typically at lower concentrations and with less consistent occurrence across samples. Notably, aflatoxin B1, a highly toxic and carcinogenic mycotoxin of particular concern in canine health, was not detected in any of the analyzed feed samples. However, sterigmatocystin, a biosynthetic precursor of aflatoxin B1, was detected in one individual sample. In addition, another *Aspergillus*-derived metabolite, kojic acid, was identified. Interestingly, plant-derived metabolites, including phytoestrogens such as daidzein, genistein, glycitein, formononetin and biochanin A were present in both cat and dog feed samples.

For some of the mycotoxins detected in the analyzed pet food samples, guidance values for critical concentrations in complete feed for dogs and cats are available. These reference thresholds were compared with the concentrations measured in our screening.

For ZEN, the guidance value is 0.1 mg/kg (100 µg/kg) of feed for both dogs and cats. The concentrations measured in the samples were 21.1 µg/kg in dog food and between 3.56 and 32.15 µg/kg in cat food, clearly below the guidance level in all cases.

For T-2 toxin and its metabolite HT-2 toxin, guidance values are 0.05 mg/kg (50 µg/kg) for cats and 0.25 mg/kg (250 µg/kg) for dogs. None of the analyzed samples, neither dog nor cat food, contained detectable amounts of these toxins.

The guidance value for fumonisins (sum of FB1 and FB2) is 5 mg/kg (5000 µg/kg) for both species. The highest concentration was found in dog food, with 303 µg/kg FB1, 84 µg/kg FB2, and 69 µg/kg FB3, resulting in a total of 456 µg/kg; this is well below 10% of the reference value. In cat food samples, total fumonisin concentrations ranged from 30 to 189 µg/kg, and FB3 was detected in only one sample. All values remained clearly below the critical threshold.

For OTA, the guidance value is 0.01 mg/kg (10 µg/kg) for both cats and dogs. OTA was detected in one cat food sample at 1.17 µg/kg, which is also clearly below the limit. In the dog food samples, OTA was not detected.

For DON, a guidance value exists only for dogs, set at 2 mg/kg (2000 µg/kg) of feed. In one of the two available dog food samples, DON was detected at a concentration of 102 µg/kg. This value is also well below the reference level. In the cat food samples, DON concentrations ranged from 89 to 154 µg/kg; however, no official guidance value exists for cats.

In summary, the detected concentrations of the investigated mycotoxins were below established guidance values in the analyzed samples; however, due to the limited number of samples, particularly for dog feed, these findings should be interpreted with caution.

### Corresponding DON and ZEN residues in feed and blood

ZEN concentrations in feline feed samples (*n* = 12) ranged from 3.56 to 32.15 µg/kg, with a mean of 16.35 µg/kg and a median of 13.79 µg/kg.

Among the tested mycotoxins (ZEN, α-ZEL, β-ZEL, ZAN, α-ZAL, β-ZAL, DON, and de-DON), only ZEN was quantifiable in feline blood samples. All other compounds were below the respective LOQs. ZEN in cat blood samples (*n* = 18) ranged from 0.04 to 0.22 ng/mL. The mean value was 0.1 ng/mL, and the median amounted to 0.09 ng/mL.

**Fig. 2:**
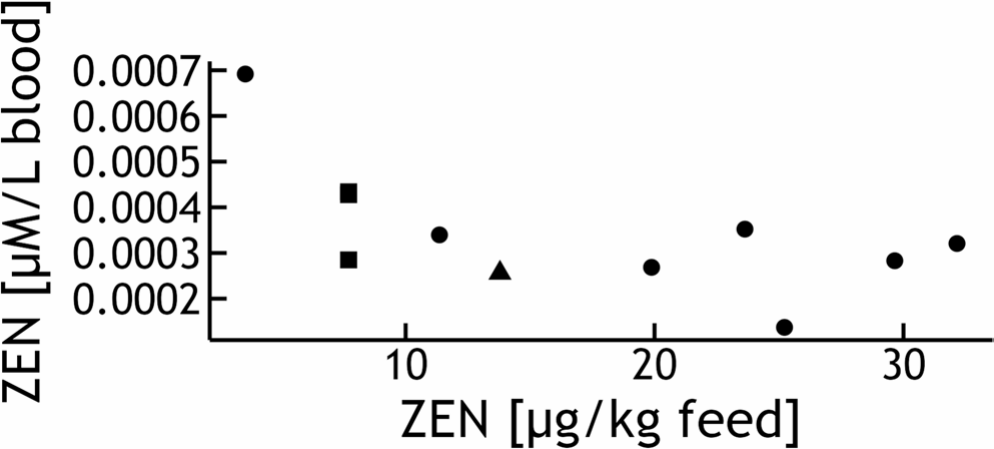
Relationship between dietary ZEN exposure in cats and corresponding individual blood concentrations. Circles represent unique feed–plasma pairs, whereas squares and triangles indicate multiple blood measurements from cats receiving the same dietary ZEN concentration (three for squares, two for triangles; partly overlapping)

To allow comparison with in vitro cytotoxicity studies, blood concentrations were converted to µM. On this basis, ZEN levels in feline blood ranged from 0.000137 to 0.000692 µM, with a mean of 0.000322 µM and a median of 0.000284 µM. An overview of these values is provided in Fig. 2, which displays blood concentrations in µM in relation to the corresponding ZEN levels in feed (*n* = 12; the feed samples shown in Fig. 2 correspond to the diets associated with the respective blood samples from the same animals).

In dogs, none of the analyzed 54 blood samples showed quantifiable levels of any of the tested toxins. This also included ZEN, for which all values remained below the LOQ. All canine blood samples originated from animals that were fed the same diet, which contained 21.1 µg ZEN/kg and thus represents background contamination. Therefore, the absence of quantifiable ZEN residues in blood is most likely the result of rapid metabolism and elimination, combined with analytical limits of quantification that are too high to capture such low-level exposure.

### In vitro cytotoxicity study

#### Four-parameter logistic (4PL) model evaluations

All combinations of species, toxins, and treatment conditions that produced poor model fits—such as non-converging models, extremely large standard errors, or implausible parameter estimates—were excluded from further 4PL analysis and were evaluated via the broken-line model instead.

**Fig. 3:**
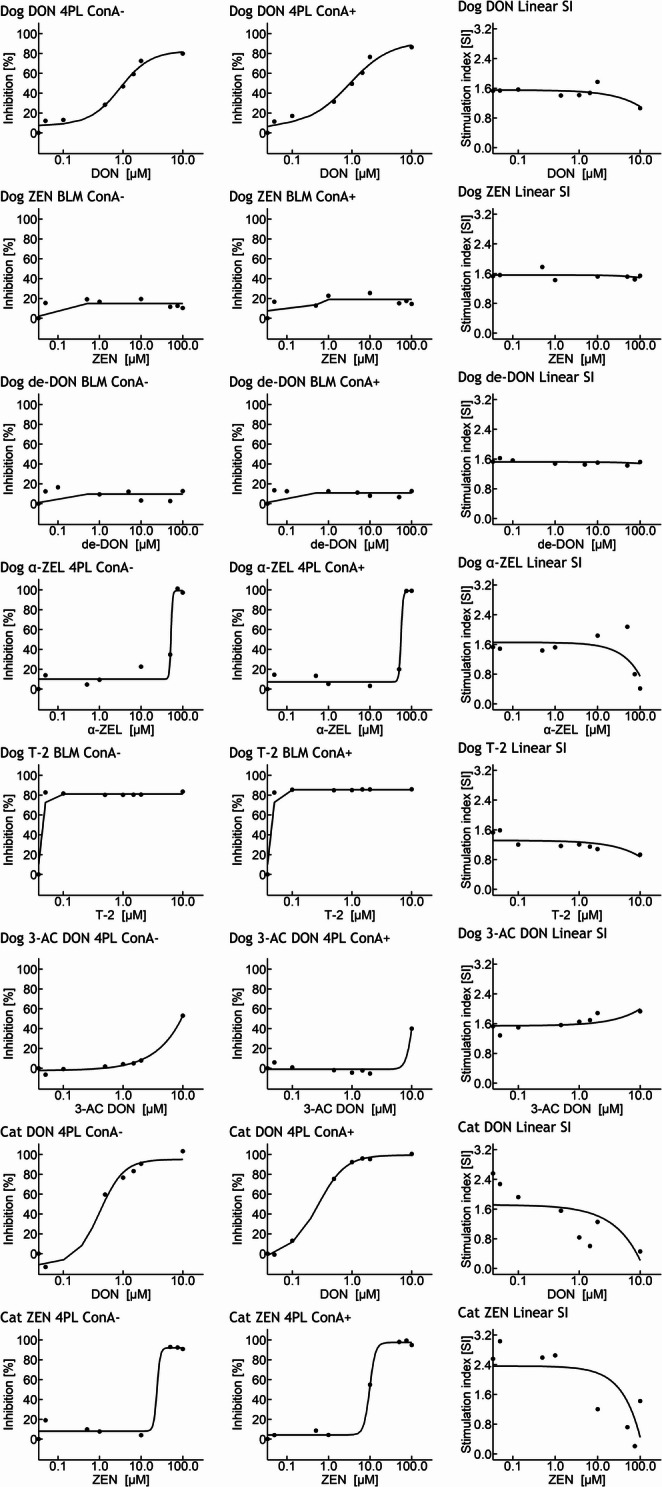
Dose–response curves for various mycotoxins in canine and feline PBMC cultures in the absence and presence of Concanavalin A (ConA). Inhibition of cell proliferation is plotted as a function of toxin concentration (left panels), and the corresponding stimulation index (SI) is shown (right panels). Data were fitted using either the four-parameter logistic model (4PL) or a broken-line model (BLM), depending on curve shape. Mycotoxins tested include deoxynivalenol (DON), 3-acetyl-DON (3-AC DON), de-epoxy-DON (de-DON), T-2 toxin (T-2), zearalenone (ZEN), and α-zearalenol (α-ZEL). Dots represent individual data points; Lines represent model fits. The absence/presence of ConA is indicated *by* ConA-/ConA+

For the remaining cases (Dog: DON, 3-AC DON, α-ZEL; each without and with ConA stimulation; Cat: DON, ZEN; each without and with ConA stimulation), the dose–response relationships between increasing concentrations of various mycotoxins and the inhibition of metabolic activity of canine and feline peripheral blood mononuclear cells (PBMCs) were successfully fitted using a four-parameter logistic regression model (Table 2; Fig. 3). The parameters estimated included the slope (b), lower and upper asymptotes (c and d), and the inflection point (e, equivalent to EC50). From these, effective concentrations at 10%, 20%, and 50% inhibition (EC10, EC20, EC50) were calculated where feasible. Model quality was assessed via the residual standard error (RSE) and the significance and precision of individual parameter estimates.

**Table 2:** Summary of 4-parameter logistic regression (4PL) results for the inhibition of metabolic activity in canine and feline PBMC by increasing mycotoxin concentrations (MTT assay), including derived effective doses (EC10, EC20, EC50) expressed as percent of untreated controls

Species	Toxin	ConA	Type	b	c	d	e	EC10	EC20	EC50	RSE
Dog	DON	-	Estimate	−1.71	7.48	82.76	0.89	0.12	0.35	1.03	5.51
			SE	0.57	3.79	6.33	0.15				
			p-value	0.04	0.12	0.00	0.00				
Dog	3-AC DON	-	Estimate	−1.05	−2.20	946.28	142.00	2.27	4.05	9.45	2.75
			SE	0.21	1.63	5055	820				
			p-value	0.01	0.25	0.86	0.87				
Dog	α-ZEL	-	Estimate	−22.84	10.07	99.34	52.15		47.61	51.67	8.84
			SE	101.96	3.95	6.27	9.86				
			p-value	0.83	0.06	0.00	0.01				
Cat	DON	-	Estimate	−2.16	−11.15	95.16	0.40	0.21	0.27	0.47	9.87
			SE	0.96	6.40	9.16	0.09				
			p-value	0.09	0.16	0.00	0.01				
Cat	ZEN	-	Estimate	−11.20	8.01	92.06	23.88	17.13	20.35	23.88	7.22
			SE	44.65	3.23	4.19	77.00				
			p-value	0.81	0.07	0.00	0.77				
Dog	DON	+	Estimate	−1.28	6.41	92.28	0.90	0.08	0.24	0.92	6.21
			SE	0.73	6.76	11.19	0.21				
			p-value	0.16	0.40	0.00	0.01				
Dog	3-AC DON	+	Estimate	−7.75	−1.01	93.19	10.34	7.97	8.80	> 10	4.64
			SE	87.57	1.75	3485	89.00				
			p-value	0.93	0.59	0.98	0.91				
Dog	α-ZEL	+	Estimate	−17.57	7.21	99.28	55.50	45.56	50.02	55.05	6.36
			SE	35.95	2.84	5.64	12.06				
			p-value	0.65	0.06	0.00	0.01				
Cat	DON	+	Estimate	−1.90	−1.82	99.48	0.27	0.09	0.14	0.27	2.26
			SE	0.16	1.93	1.59	0.02				
			p-value	0.00	0.40	0.00	0.00				
Cat	ZEN	+	Estimate	−6.34	4.15	97.43	9.73	6.35	7.58	9.68	3.43
			SE	45.79	1.72	1.98	1.92				
			p-value	0.90	0.07	0.00	0.01				

Estimated and derived parameters of the 4PL *model b *= slope (unitless), *c* = minimum response (%), *d* = maximum response (%), *e* = concentration at 50% effect between c and d (µM), *EC10/20/50* = effective concentration for 10%/20%/50% inhibition (µM), *RSE* = residual standard error (%), *SE* = standard error, *ConA* = concanavalin A, *DON* = deoxynivalenol, *α-ZEL* = alpha-zearalenol, *ZEN* = zearalenone, *3-AC DON* = 3-acetyl-deoxynivalenol

In canine PBMCs without ConA stimulation, DON produced a well-fitting curve (RSE = 5.51) with an upper asymptote (d) of 82.8%, indicating a maximum inhibition of approximately 83% compared to untreated controls. The EC50 was 1.03 µM, while EC10 and EC20 were 0.12 and 0.35 µM, respectively. Slope (b), maximum response (d) and concentration at 50% effect between c and d (e) were statistically significant while the lower asymptote (c) failed reaching significance (Table 2).

For 3-acetyl-DON (3-AC DON) in unstimulated canine PBMCs, the logistic model converged with a relatively low residual error (RSE = 2.75), and EC values (EC10 = 2.27 µM; EC20 = 4.05 µM; EC50 = 9.45 µM) were within the same range as those observed under ConA-stimulated conditions. However, the upper asymptote estimate (d = 946.3%) was biologically implausible and associated with an extremely large standard error (SE = 5055), suggesting that the model overestimated the maximal inhibition due to the absence of a clearly defined plateau. Despite this, the slope parameter (b) was statistically significant (*p* = 0.01), and the EC estimates may still provide a reasonable approximation of the concentration–response relationship. Nonetheless, results should be interpreted with caution due to the poor reliability of the upper asymptote.

For α-zearalenol (α-ZEL), the estimated maximum inhibition was higher, at 99.3% (d), but with greater uncertainty in the slope (b) and its large standard error (SE = 102), suggesting a flat or unstable curve. The EC50 was 51.7 µM, with EC20 and EC10 estimated at 47.6 and 0 µM (not defined or unreliable).

Under ConA-stimulated conditions, DON again resulted in a good model fit in canine PBMCs (RSE = 6.21) with a slightly higher maximum inhibition of 92.3%. The EC50 was 0.92 µM, and EC10 and EC20 were 0.08 and 0.24 µM, respectively. While most parameters were well estimated, the slope and lower asymptote showed less certainty.

For 3-acetyl-DON (3-AC DON), although the model technically converged (RSE = 4.64), the upper asymptote had an extremely large standard error (SE = 3484.5), and none of the estimated parameters reached significance. The EC50 was only reported as greater than 10 µM, indicating a poor and unreliable fit.

α-ZEL showed a similarly high maximum inhibition (99.3%), and EC values (EC10 = 45.6 µM; EC50 = 55.1 µM) were consistent with the unstimulated condition. However, the slope parameter was not statistically significant.

In feline PBMCs without ConA, DON also resulted in a successful fit (RSE = 9.87) with a maximum inhibition of 95.2%. The EC50 was relatively low at 0.47 µM, and EC10 and EC20 were 0.21 and 0.27 µM, respectively. While the upper asymptote and inflection point were estimated with precision, the slope and lower asymptote showed less reliability. Zearalenone (ZEN) produced a fit of moderate quality (RSE = 7.22) with a maximum inhibition of 92.1%. The EC50 was 23.9 µM, while EC10 and EC20 were 17.1 and 20.4 µM, respectively. However, only the upper asymptote reached statistical significance, and the remaining parameters were affected by high standard errors.

In ConA-stimulated feline PBMCs, DON again showed a robust fit with low residual error (RSE = 2.26) and a high maximum inhibition of 99.5%. All model parameters were highly significant and precisely estimated. The EC values were 0.09 µM (EC10), 0.14 µM (EC20), and 0.27 µM (EC50), indicating a steep and consistent inhibition curve. ZEN, under the same conditions, also showed a good fit (RSE = 3.43), with a maximum inhibition of 97.4%. The derived EC10, EC20, and EC50 were 6.35, 7.58, and 9.68 µM, respectively. Most parameters were statistically significant or close to significance, with relatively small standard errors.

Overall, maximal inhibition levels (d) across the reliable models ranged from 82.8% to 99.5%, with most mycotoxins reaching near-complete inhibition in at least one condition. However, the slope parameter (b) was occasionally unstable or non-significant, particularly for α-ZEL and 3-AC DON in dogs, suggesting potential limitations in curve steepness or transition zone definition. In particular, results for 3-AC DON in dogs should be interpreted with caution due to the extremely poor reliability of parameter estimation, despite apparent convergence.

#### Broken-line model (BLM) evaluations

Dose–response data for selected mycotoxins were additionally modeled using the broken-line model (BLM), which estimates an initial linear increase in inhibition up to a breakpoint concentration, after which the response reaches a plateau. For canine PBMCs, the BLM was applied to T-2 toxin, de-DON, and ZEN, both in the presence and absence of ConA stimulation (Table 3; Fig. 3).

**Table 3:** Estimated parameters of the broken-line model (BLM) describing the concentration–response relationship of selected mycotoxins in canine PBMCs, with and without ConA stimulation

Species	Toxin	ConA	Type	intercept	slope	constant	RSE	breakpoint
Dog	ZEN	-	Estimate	2.12	223	14.90	4.19	0.06
			SE	4.19	119	1.71		
			p-value	0.63	0.12	0.00		
Dog	de-DON	-	Estimate	1.07	172	9.67	5.72	0.05
			SE					
			p-value					
Dog	T-2	-	Estimate	10.38	1240	81.1	6.69	0.06
			SE	6.69	189	2.73		
			p-value	0.18	0.00	0.00		
Dog	ZEN	+	Estimate	7.62	11.92	19.02	6.67	0.96
			SE	4.97	17.14	2.98		
			p-value	0.19	0.52	0.00		
Dog	de-DON	+	Estimate	1.27	192	10.83	2.95	0.05
			SE					
			p-value					
Dog	T-2	+	Estimate	10.10	1249	85.5	6.40	0.06
			SE	6.40	181	2.61		
			p-value	0.18	0.00	0.00		

*Intercept *= model intercept on y-axis (%), *slope* = rate of increase before breakpoint (%/µM), *constant* = plateau response after breakpoint (%), *breakpoint* = concentration at which the slope transitions to a constant (µM), *RSE* = residual standard error (%), *SE* = standard error, *ConA* = concanavalin A, *de-DON* = de-epoxy-deoxynivalenol, *ZEN* = zearalenone; T-2 = T-2 toxin

For certain model variants, standard errors and p-values could not be computed due to numerical limitations inherent in the iterative estimation procedure. These issues likely stem from near-singular Hessian or variance-covariance matrices, which may arise when the model is overparameterized, poorly conditioned, or when parameters are not identifiable from the data. Consequently, although point estimates were obtained, statistical inference (SE and p-value) was not possible in these cases

In the absence of ConA, T-2 showed a steep linear increase in inhibition (slope = 1240) up to a breakpoint of 0.06 µM, after which the response plateaued at 81.11%. The model fit was acceptable (RSE = 6.69), and both the slope and plateau were statistically significant (*p* < 0.001). de-DON showed a more moderate response, reaching a constant inhibition level of 9.67% after a breakpoint at 0.05 µM, though no standard errors or p-values were available for this fit. For ZEN, the model estimated a breakpoint at 0.06 µM and a plateau at 14.90%; however, only the plateau estimate reached statistical significance.

Under ConA-stimulated conditions, similar patterns were observed. T-2 again exhibited a very steep increase (slope = 1249) up to a breakpoint of 0.06 µM, with a plateau at 85.5% and a good model fit (RSE = 6.40). de-DON reached a slightly higher plateau (10.83%) at a comparable breakpoint of 0.05 µM. For ZEN, a delayed breakpoint was observed at 0.96 µM, and the plateau was estimated at 19.02%; however, neither the intercept nor slope was statistically significant, while the plateau was again highly significant (*p* < 0.001).

Overall, the BLM captured the early-phase increase in inhibition followed by saturation for these compounds. T-2 consistently showed the steepest response, while ZEN and de-DON produced more moderate effects with lower maximal inhibition levels.

The very high slope estimates observed in some models (e.g., T-2 toxin) are likely a consequence of the data distribution and the early breakpoint, leading to steep increases over a narrow concentration range. These estimates should therefore be interpreted with caution, as they may overestimate the actual biological response.

#### Summary of estimated EC50 values

Table 4 provides a comparative overview of EC50 values derived from the dose–response analyses presented above. These values originate from fits using either the four-parameter logistic model (4PL) or, where appropriate, the broken-line model (BLM). The 4PL model was applied to data sets showing a sigmoidal inhibition profile with clear lower and upper asymptotes. In contrast, the BLM was used in cases where the response increased approximately linearly and then reached a plateau, without exhibiting a classical sigmoidal shape. For these cases, the estimated breakpoint describes the concentration at which the model shifts from a linear increase to a constant response. While this breakpoint does not correspond to the EC50 in the strict sense, it offers an approximate indicator of the transition point beyond which further increases in toxin concentration no longer enhance inhibition.

**Table 4:** Summary of estimated EC50 values (µM) for various mycotoxins in canine and feline PBMCs in the presence or absence of ConA

Species	Toxin	ConA	EC50 [µM]	Comment
Dog	DON	–	1.03	Good model fit (RSE 5.51)
Dog	DON	+	0.92	Good model fit (RSE 6.21)
Dog	3-AcDON	–	9.45	Plateau > 50%; unusually steep curve (slope 946%)
Dog	3-AcDON	+	> 10.00	Plateau < 50%; no 50% inhibition
Dog	de-DON	–	> 100.0	Plateau at 9.7%; EC50 not reached at 100 µM
Dog	de-DON	+	> 100.0	Plateau at 10.8%; EC50 not reached at 100 µM
Dog	T-2	–	< 0.10	Strong inhibition at lowest tested concentration
Dog	T-2	+	< 0.10	Strong inhibition at lowest tested concentration
Dog	ZEN	–	> 100.0	Plateau at 14.9%; EC50 not reached at 100 µM
Dog	ZEN	+	> 100.0	Plateau at 19.0%; EC50 not reached at 100 µM
Dog	α-ZEL	–	51.70	High SE; uncertain fit
Dog	α-ZEL	+	55.10	Slope not significant; flat dose–response curve
Cat	DON	–	0.47	Good fit; low residual error (RSE 9.87)
Cat	DON	+	0.27	Very good fit; all parameters significant (RSE 2.26)
Cat	ZEN	–	23.90	Only plateau significant; high variability in slope/intercept
Cat	ZEN	+	9.68	Most parameters significant; reliable estimate

*ConA* concanavalin A, *EC50* effective concentration at 50% inhibition, *SE* standard error, *RSE* residual standard error (µM), DON, deoxynivalenol, *3-AcDON* 3-acetyl-deoxynivalenol, *de-DON* de-epoxy-deoxynivalenol, *T-2* T-2 toxin, *ZEN* zearalenone, *α-ZEL* alpha-zearalenol

EC50 values (in µM) were calculated from four-parameter log-logistic (4PL) or broken-line models (BLM) fitted to the dose–response curves of peripheral blood mononuclear cells (PBMCs) from dogs and cats exposed to various mycotoxins in the presence or absence of ConA. Where the estimated plateau was below 50%, or inhibition exceeded 50% at the lowest or highest tested dose, EC50 values were reported as being outside the tested range

This summary facilitates the comparison of immunomodulatory potency across different mycotoxins, stimulation conditions (± ConA), and species. However, biological relevance of the EC50 values should be interpreted with caution, particularly in cases where the maximum inhibition remains below 50%, or where the estimated EC50 lies outside the experimentally tested concentration range.

#### Stimulation indices

To assess the effects of various mycotoxins on the responsiveness of PBMC, linear regressions were performed using the stimulation index as the dependent variable and toxin concentration as the independent variable (Table 5; Fig. 3).

**Table 5:** Linear regression parameters describing the effect of increasing mycotoxin concentrations on the stimulation index in canine and feline PBMC cultures

Species	Toxin	Type	intercept	slope	RSE
Dog	DON	Estimate	1.55	−0.04	0.14
		SE	0.06	0.02	
		p-value	0.00	0.03	
Dog	ZEN	Estimate	1.56	0.00	0.11
		SE	0.05	0.00	
		p-value	0.00	0.52	
Dog	de-DON	Estimate	1.52	0.00	0.07
		SE	0.03	0.00	
		p-value	0.00	0.56	
Dog	α-ZEL	Estimate	1.66	−0.01	0.43
		SE	0.19	0.00	
		p-value	0.00	0.06	
Dog	T-2	Estimate	1.32	−0.04	0.18
		SE	0.07	0.02	
		p-value	0.00	0.07	
Dog	3-AC DON	Estimate	1.54	0.05	0.16
		SE	0.06	0.02	
		p-value	0.00	0.04	
Cat	DON	Estimate	1.72	−0.15	0.64
		SE	0.27	0.07	
		p-value	0.00	0.08	
Cat	ZEN	Estimate	2.37	−0.02	0.77
		SE	0.34	0.01	
		p-value	0.00	0.04	

*DON* deoxynivalenol, *ZEN* zearalenone, *de-DON* de-epoxy-deoxynivalenol, *α-ZEL* alpha-zearalenol, *T-2* T-2 toxin, *3-AC DON* 3-acetyl-deoxynivalenol, *PBMC* peripheral blood mononuclear cells, *SE* standard error, *RSE* residual standard error

In samples derived from dogs, exposure to deoxynivalenol (DON) resulted in a significant negative association between stimulation index and toxin concentration (slope: − 0.04; *p* = 0.03). In contrast, exposure to ZEN and de-epoxy-DON showed no statistically significant trends (*p* = 0.52 and *p* = 0.56, respectively). For α-ZEL, a negative trend was observed (slope: − 0.01; *p* = 0.06). Similarly, T-2 toxin demonstrated a negative trend in slope (slope: − 0.04; *p* = 0.07). In contrast, exposure to 3-acetyl-DON showed a statistically significant positive slope (slope: 0.05; *p* = 0.04).

In samples from cats, exposure to DON resulted in a negative trend in slope (slope: − 0.15; *p* = 0.08), whereas exposure to ZEN yielded a statistically significant negative slope (slope: − 0.02; *p* = 0.04).

Although some associations reached statistical significance, the estimated slopes were generally small, indicating limited effect sizes. Moreover, the relatively high residual standard errors suggest considerable variability in the data, and thus a limited explanatory power of the linear models. Nevertheless, the consistent direction of effects observed for certain toxins (e.g., DON) may indicate biologically relevant trends that warrant further investigation.

## Discussion

### Feed screening

In addition to mycotoxins, the applied multi-analyte LC-MS/MS method also detects a wide range of naturally occurring plant and microbial metabolites. Although these compounds were not the primary focus of the study, their presence provides additional information on the compositional characteristics of the analyzed feed matrices and may support the interpretation of differences between feed categories.

The comparative assessment of metabolite profiles in different dog and cat feed categories revealed substantial variation in concentration patterns across metabolite groups. Although the dataset for certain specialized feeds was limited to single samples, descriptive tendencies were apparent. Overall, metabolite concentrations spanned several orders of magnitude, with most compounds present across multiple feed types, and differences arising primarily from concentration levels rather than presence or absence.

*Fusarium*-derived metabolites, including enniatins, beauvericin, DON, and 15-hydroxyculmorin, were more frequently detected at elevated concentrations in “other cat feed” and “all dog feed” compared with the senior and intestinal cat feeds, which consistently showed lower levels. Such reductions may indicate a reduced maize and wheat content, as these cereals are common sources of *Fusarium* toxin contamination (European Food Safety Authority 2014; Schatzmayr and Streit 2013; Streit et al. 2012). Similarly, other fungal metabolites displayed higher concentrations in standard feeds than in specialized products. Despite their relatively high occurrence in the investigated pet food samples, the toxicological relevance of these mycotoxins, particularly with respect to immune cell toxicity, remains largely unexplored in dogs and cats. Terpestacin (siccanol), a deacetylated intermediate in the fusaproliferin biosynthetic pathway, interferes with mitochondrial function and modulates ROS-dependent signaling pathways (Jung et al. 2010). Its acetylated derivative fusaproliferin exhibits cytotoxic effects in mammalian cells, including human B lymphocytes, with reported IC50 values of approximately 50–70 µM (Logrieco et al. 1996; Ritieni et al. 1995). In contrast, culmorin and its hydroxylated derivatives show little to no intrinsic cytotoxicity in mammalian cell systems (IC50 > 100 µM or not reached), and no specific lymphocyte toxicity has been demonstrated; however, culmorin can inhibit UDP-glucuronosyltransferase-mediated detoxification of deoxynivalenol, thereby potentially increasing systemic exposure to co-occurring mycotoxins(Weber et al. 2018; Woelflingseder et al. 2019). The cyclic depsipeptide beauvericin, by contrast, exhibits pronounced cytotoxicity in mammalian cells, including lymphoid cell systems, with IC50 values typically in the low micromolar range (~ 1–5 µM), mediated by its ionophoric properties, calcium dysregulation, and apoptosis induction (Jestoi 2008; Mallebrera et al. 2018). Given the central role of mitochondrial function, ion homeostasis, and xenobiotic metabolism in lymphocyte viability, these co-occurring Fusarium metabolites could hypothetically contribute to altered immune cell responses in dogs and cats under conditions of combined exposure; however, direct species-specific evidence is currently lacking.

Phytoestrogens such as secoisolariciresinol and formononetin tended to be more abundant in “other cat feed” and “all dog feed,” whereas “cat indoor feed” displayed lower levels. This pattern may reflect differences in the inclusion of phytoestrogen-rich raw materials such as soy and linseed, which are known to contain phytoestrogen precursors including isoflavones and lignans (Murkies et al. 1998). Plant metabolites, notably abscisic acid, were also found at higher levels in standard cat and dog feeds, potentially indicating a greater proportion of plant-based components such as vegetables, fruits, and legumes although this classical phytohormone was also described to be synthesized by organisms others than plants including mammals (Li et al. 2011). In contrast, specialized feeds tended to show reduced levels in this metabolite group, possibly due to a higher proportion of animal-derived ingredients or selective raw material sourcing.

*Penicillium* toxins occurred at generally low to moderate levels, with only minor category differences, and Aspergillus and *Alternaria* toxins were detected at low levels across all feed types, suggesting minimal influence of these toxin groups on feed-type differentiation. Unspecific metabolites showed a mixed pattern, with higher concentrations in “all dog feed” and, to a lesser extent, “other cat feed.”

**Fig. 4:**
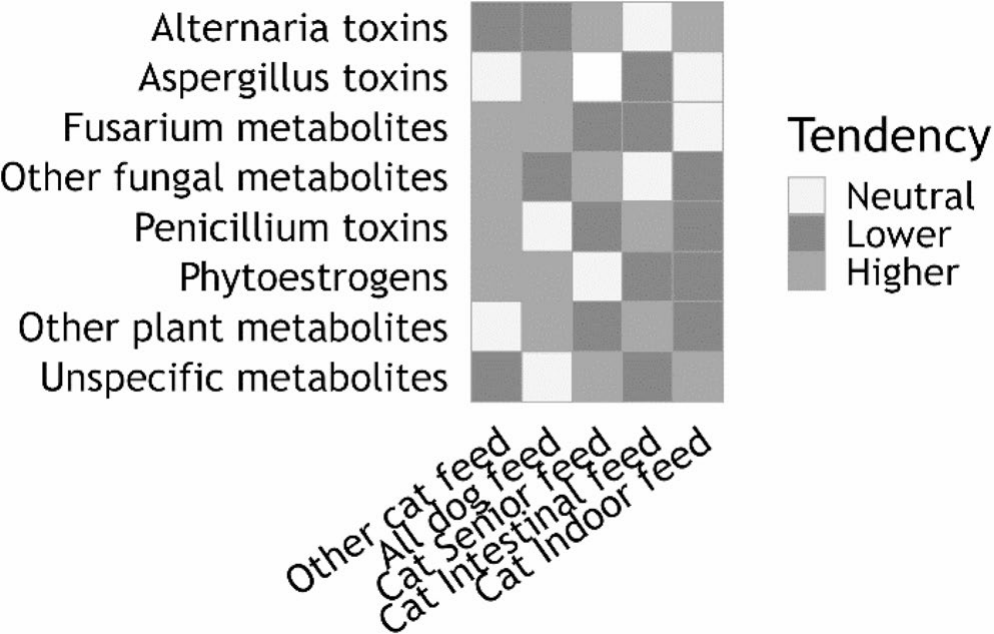
Heatmap showing relative tendencies of metabolite groups across feed types. Grading (Lower, Neutral, Higher) was derived within each toxin group by comparing feed-specific medians to an overall, sample-size-weighted median and classifying weighted log-ratio effects using group-specific quantile thresholds

In summary, standard feed types for both cats and dogs appear more likely to exhibit elevated concentrations in several metabolite groups, particularly those associated with cereal-based or plant-rich formulations. Specialized feeds, particularly senior and intestinal formulations, consistently presented lower concentrations across multiple metabolite categories, potentially due to targeted ingredient selection and processing to meet specific nutritional or health-related goals (Fig. 4).

In conclusion, the feed metabolome primarily reflects the composition of the raw materials used rather than allowing a direct prediction of biological effects. For those mycotoxins with established guidance values, the measured concentrations indicated no toxicological concern under the tested conditions. However, for most other detected metabolites, including potential interactions between them, toxicological relevance remains largely unknown—particularly for companion animal species such as cats and dogs.

### Cytotoxicity of mycotoxins to PBMC and relation to internal exposure

Previous in vitro studies have demonstrated the immune-toxic potential of various mycotoxins in leukocytes. Given the limited availability of studies specifically addressing canine and feline PBMCs, selected studies on structurally and toxicologically related mycotoxins were included to provide general mechanistic context rather than direct comparability.

In dogs, exposure of leukocytes from healthy young animals (1–2 years old, Persian/herd breeds) to aflatoxin B1 at 10 ng/mL (~ 0.032 µM) for 24 h caused a marked reduction in intracellular ATP (− 33.2 ± 2.7%) and more than a twofold increase in caspase-3/7 activity across neutrophils, lymphocytes, and monocytes, accompanied by a slight, non-significant increase in necrotic cells (Mehrzad et al. 2020). Although aflatoxin B1 was not part of the present PBMC exposure experiments, it was included in the analytical screening of feed samples (not detected) and is considered here to illustrate general leukocyte responses to potent mycotoxins. In a separate study, Singh et al. (2018) treated canine PBMCs with extracts of commercial dog food containing multiple mycotoxins (AFB1, FB1, OTA, and ZEN), which were standardized by HPLC to a total combined mycotoxin concentration of 40 µg/mL in the extract. Although the concentrations of individual mycotoxins were not reported, an equimolar assumption suggests an average molar concentration of ~ 25 µM. Both standard-brand (SB) and premium-brand (PB) extracts induced significant oxidative stress, mitochondrial dysfunction, apoptosis, and necrosis, with SB extracts causing greater cell damage than PB extracts. A similar approach in cats (Singh et al. 2020) yielded comparable cytotoxic profiles, with PB extracts again showing milder effects than SB despite measurable immunotoxicity in both.

When these mixture-exposure results are compared to our single-compound EC50 values, clear differences emerge. For ZEN, no 50% inhibition of canine PBMC proliferation was observed at concentrations up to 100 µM, irrespective of ConA stimulation, whereas feline PBMCs showed EC50 values of 23.9 µM (− ConA) and 9.68 µM (+ ConA). The estimated ~ 25 µM total molar concentration in the mixture extracts is therefore well below the canine EC50 for ZEN and only at or slightly above the feline EC50. This suggests that ZEN alone was not responsible for the observed cytotoxicity in the studies by Sing et al. (2018), and that co-occurring toxins with higher potency—such as aflatoxin B1, fumonisin B1, or ochratoxin A—are plausible contributors to the observed effects; however, it remains unclear whether these effects arise from individual toxins or from combined (additive or synergistic) interactions. The relative potency ranking in our EC50 data is consistent with reports from other immune cell models including peripheral primary PBMC, with T-2 toxin showing extreme potency (EC50 < 0.1 µM), DON showing moderate potency (sub-µM to low µM, species-dependent), and de-epoxy DON derivatives as well as ZEN and alpha-ZEL as markedly less toxic (Dänicke and Winkler 2015; European Food Safety Authority 2017; Schumann et al. 2016).

The interpretation of these in vitro thresholds must be considered in the context of actual internal exposure under practical feeding conditions. In feline feed samples (*n* = 12), ZEN concentrations ranged from 3.56 to 32.15 µg/kg (mean 16.35 µg/kg), yet only ZEN was quantifiable in feline blood, with concentrations between 0.0436 and 0.22 ng/mL (0.000137–0.000692 µM; mean 0.000322 µM). These levels are roughly four orders of magnitude lower than the EC50 values determined in the present study. In dogs, ZEN was not quantifiable in any of the 54 blood samples analyzed, including those corresponding to feed samples with measurable ZEN (21.1 µg/kg). For DON, all blood values in both species remained below the respective limits of quantification. These findings indicate that, under the tested feeding scenarios, systemic exposure to ZEN and DON is unlikely to reach levels capable of inducing direct cytotoxic effects in PBMC, even in the more sensitive feline model.

### Model-based parameter interpretation

Analysis of parameters derived from the BLM revealed distinct differences in immunotoxic potency among the tested mycotoxins. T-2 toxin consistently exhibited strong inhibitory effects, with EC50 values below the lowest tested concentration (< 0.1 µM), indicating high toxicity toward canine PBMCs. In contrast, de-DON and ZEN showed only limited inhibitory potential, with plateau values well below 50% and EC50 values exceeding the highest tested concentration (> 100 µM). The presence of ConA modulated sensitivity in a toxin-specific manner, enhancing the plateau response for ZEN but having little to no impact on de-DON. These results highlight the value of the BLM for identifying concentration thresholds (breakpoints) and differentiating between toxins with sharp dose–response transitions and those with gradual or incomplete inhibition curves.

### Stimulation index

Beyond EC50 estimation, the stimulation index (SI), defined as the ratio of ConA-stimulated to unstimulated PBMC responses, provided additional insight into lymphocyte responsiveness under mitogen stimulation and allowed indirect comparison of ConA + and ConA− conditions. Overall, the relatively moderate differences between ConA + and ConA− conditions indicate that the tested mycotoxins affected both baseline and mitogen-stimulated responses in a comparable manner. Several compounds, most notably DON in dogs (*p* = 0.03) and ZEN in cats (*p* = 0.04), significantly decreased SI with increasing concentration, suggesting that certain mycotoxins impair lymphocyte responsiveness to polyclonal stimuli. Trends toward reduced stimulation were also observed for T-2 (*p* = 0.07) and alpha-ZEL (*p* = 0.06) in canine PBMCs. In contrast, 3-acetyl-DON in dogs showed a significant positive slope (*p* = 0.04), potentially indicating a biphasic or hormetic response, suggesting a differential modulation of stimulated versus basal activity. This pattern differs from DON and could be related to differences in cellular uptake or metabolic conversion, although the underlying mechanisms remain to be elucidated. No clear correlation was found between EC50 values and SI slopes, reflecting that these parameters capture distinct aspects of immune modulation.

### Species-specific differences in PBMC responsiveness to DON and ZEN

Among the tested mycotoxins, only DON and ZEN were evaluated in both canine and feline PBMC cultures, allowing direct interspecies comparison. DON induced stronger proliferation inhibition in feline PBMCs than in canine cells, and ZEN showed an even more pronounced species-specific difference, with marked inhibition in feline PBMCs but limited effects in dogs. These differences may reflect species-specific xenobiotic metabolism. Cats have a reduced glucuronidation capacity due to deficiencies in several UDP-glucuronosyltransferase (UGT) enzymes (Court 2013), which may slow detoxification and prolong cellular exposure to active ZEN or its metabolites. In contrast, dogs possess a broader repertoire of these enzymes, although such species differences are substrate-dependent (Court 2013). While species differences in xenobiotic metabolism, particularly in hepatic phase II pathways, are well documented, it should be noted that PBMCs exhibit only limited metabolic capacity compared to liver tissue. Although PBMCs possess xenobiotic-metabolizing enzymes, their metabolic capacity is markedly reduced compared to hepatic tissue, with only a limited subset of enzymes being expressed and no consistent correlation to liver expression levels (Furukawa et al. 2004). Therefore, PBMCs cannot be considered a functionally equivalent metabolic system to the liver.

Importantly, phase II metabolism (e.g., glucuronidation or sulfation) generally reduces the biological activity and cytotoxicity of mycotoxins by increasing their polarity and limiting cellular uptake. This is supported by in vitro data for DON, where the parent compound exhibits pronounced cytotoxicity (IC50 ≈ 1.3 µM), whereas its major metabolite, DON-glucuronide, shows no significant cytotoxicity even at concentrations up to 270 µM (Wu et al. 2007). Furthermore, DON-glucuronide did not modulate DON-induced toxicity, confirming its role as a detoxification product.

Consequently, the cytotoxic effects observed in PBMCs are likely primarily driven by the parent compounds, while species-specific differences in metabolic capacity may contribute to variability in cellular sensitivity to a limited extent. ZEN residues in blood further support this interpretation. While dietary ZEN exposure levels were comparable, ZEN was detected only in feline blood and not in canine samples, although this may partly reflect analytical sensitivity, as the LOQ for ZEN in dog plasma and serum was higher (0.80–1.03 ng/mL) than in feline serum (0.07 ng/mL). Consequently, ZEN concentrations in dog blood may have been within the same range as in cats but remained undetectable. Future studies with more sensitive analytical methods are warranted to clarify whether these findings reflect true toxicokinetic differences or methodological constraints.

## Conclusions

The contamination levels in the examined cat and dog foods were within background ranges or remained undetectable and did not exceed guidance values or maximum limits for AFB1, DON, ZEN, OTA, FB1 + FB2, and T-2/HT-2 toxins. For other detected mycotoxins, no risk evaluation could be made due to insufficient toxicological data for companion animals. The observed cytotoxicity patterns align with potency rankings from other species, and clear interspecies differences in PBMC sensitivity to DON and ZEN were observed, with feline PBMCs responding more sensitively. Importantly, blood concentrations of DON and ZEN were several orders of magnitude lower than the in vitro EC50 values, suggesting that under practical feeding conditions, direct cytotoxic thresholds are unlikely to be reached. Further screening studies are needed to better characterize pet food contamination in relation to composition and to refine toxicological risk assessments for companion animals.

Overall, these findings indicate that, under the conditions investigated, a relevant health risk due to mycotoxin exposure via commercial pet food appears unlikely. However, the presence of multiple mycotoxins at low levels highlights the need for continued monitoring and consideration of potential additive or combined effects.

## Data Availability

The data supporting the findings of this study are available from the corresponding author upon reasonable request.
